# Longitudinal trends (2011–2020) of premature mortality and years of potential life loss (YPLL) and associated covariates of the 62 New York State counties

**DOI:** 10.1186/s12939-023-01902-w

**Published:** 2023-05-16

**Authors:** Maria Isabel Roldós, John Orazem, Talita Fortunato-Tavares

**Affiliations:** 1 Department of Health Equity, Administration and Technology, School of Health Sciences, Human Services and Nursing, New York, USA; 2 City University of New York (CUNY) Institute for Health Equity, New York, USA; 3 Lehman College, City University of New York (CUNY), New York, USA; 4 Department of Speech-Language Hearing Sciences, School of Health Sciences, Human Services and Nursing, New York, USA; 5 Affiliated faculty, CUNY Institute for Health Equity, New York, NY USA

**Keywords:** County rankings, Longitudinal, New York State, Clusters

## Abstract

**Background:**

New York State (NYS) is the 27^th^ largest state and the 4^th^ most populous state in the U.S., with close to 20 million people in 62 counties. Territories with diverse populations present the best opportunity to study health outcomes and associated covariates, and how these differ across different populations and groups. The County Health Ranking and Roadmaps (CHR&R) ranks counties by linking the population’s characteristics and health outcomes and contextual factors in a synchronic approach.

**Methods:**

The goal of this study is to analyze the longitudinal trends in NYS counties of age-adjusted premature mortality rate and years of potential life loss rate (YPLL) from 2011–2020 using (CHR&R) data to identify similarities and trends among the counties of the state. This study used a weighted mixed regression model to analyze the longitudinal trend in health outcomes as a function of the time-varying covariates and clustered the 62 counties according to the trend over time in the covariates.

**Results:**

Four clusters of counties were identified. Cluster 1, which represents 33 of the 62 counties in NYS, contains the most rural counties and the least racially and ethnically diverse counties. Clusters 2 and 3 mirror each other in most covariates and Cluster 4 is comprised of 3 counties (Bronx, Kings/Brooklyn, Queens) representing the most urban and racial and ethnic diverse counties in the state.

**Conclusion:**

The analysis clustered counties according to the longitudinal trends of the covariates, and by doing so identified clusters of counties that shared similar trends among the covariates, to later examine trends in the health outcomes through a regression model. The strength of this approach lies in the predictive feature of what is to come for the counties by understanding the covariates and setting prevention goals.

**Supplementary Information:**

The online version contains supplementary material available at 10.1186/s12939-023-01902-w.

## Introduction

New York State (NYS) is the 27^th^ largest state and the 4^th^ most populous state in the U.S., with close to 20 million people in 62 counties [1]. The most recent NYS’s Health Equity County Report compared various diseases and illnesses among racial and ethnic minorities and found that 19% of non-Hispanic African American or Black families and 23,4% of Hispanic families lived in poverty compared to 6,4% White non-Hispanic families. Furthermore, Non-Hispanic African American or Blacks had the highest age-age-adjusted total mortality rate compared to all other racial and ethnic groups with a rate of 695,1 per 100,000, followed by Hispanics with a total age-adjusted mortality rate of 493,2 per 100,000 [2]. Morbidity outcomes such as age-adjusted asthma hospitalization rates among non-Hispanics Blacks were 38 per 10,000 and 28,0 per 10,000 for Hispanics compared to 7,3 per 10,000 White non-Hispanics [2]. In addition, New York City (NYC) Hispanic residents have the highest age-adjusted rates of diabetes mortality within the state, with rates of 21,4 per 100,000 and hospitalizations during 2014–2016 [3].

Territories with diverse populations present the best opportunity to study health outcomes and how these differ across different populations and groups [4]. Furthermore, longitudinal trends draw attention to the role of context, including social and physical environments that may underlie socioeconomic, racial, and ethnic health disparities [5–7]. Best practices in health disparities research (HDR) suggest higher incidence or prevalence of illness, injuries, and premature mortality as the health outcomes to measure health disparities, and social determinants of health (SDOH) frameworks to understand the causes of these disparities [8, 9].

SDOH‐based research allows studying the influence of socioeconomic status, socio-behavioral, cultural, community and environment to various outcomes [10]. Studying the role of social determinants associated with a specific health outcome between populations and territories has the potential to identify populations at greater risk in relation to their location [11, 12]. For example, studying the role of social determinants associated with cardiovascular disease (CVD) outcomes from 2009 to 2018 in all U.S. counties suggested that over a 10‐year period, CVD mortality declined at an annual rate of 1.08 (95% CI, 0.74–1.42) deaths per 100 000 people, and rural counties and counties with a higher percentage of non-Hispanic Black residents had a consistently higher CVD mortality rate than urban counties and counties with a lower percentage of non-Hispanic Black residents [10]. Another study that investigated county-level mortality data related to COVID 2020–2021 within U.S. sought to understand county-level variation [13]. The results suggested that mortality disparities are not driven by fixed county-level characteristics or changes in the regional dispersion of COVID-19, but instead by changes within counties.

Since 2010, the University of Wisconsin Population Health Institute and the Robert Wood Johnson Foundation have produced the County Health Rankings and Roadmaps (CHR&R) [14]. The CHR&R project yearly ranks nearly every county of the U.S. in relation to health outcomes and associated factors. New York State’s ranking for 2023 had Putnam, Saratoga, Nassau, Rockland and Tompkins counties ranked as the healthiest counties in the state, and Chenango, Montgomery, Chemung, Sullivan, Cattaraugus and Bronx as the least healthy counties [15]. The CHR&R cross-sectional approach correlated the population’s characteristics and contextual factors with health outcomes in a synchronic approach. Results from the yearly rankings do not identify similarities between counties nor does it detail the trends in any specific factor or social determinant.

The goal of this study is to analyze the longitudinal trends in NYS counties of age-adjusted premature mortality rate and years of potential life loss rate (YPLL) from 2011–2020 using the County Health Ranking and Roadmaps (CHR&R) data to identify similarities and trends among the counties of the state.

## Methods

### Dataset

This is a secondary data analyses project using data from the County Health Rankings and Roadmaps CHR&R data set [16]. CHR&R data and project is based on a model of population health that emphasizes the many social, economic, physical, clinical, and other factors that influence health outcomes and multiple covariates factors [14]. CHR&R describes the county’s health state into two components; length of life (including premature death, life expectancy and infant mortality), and quality of life (including self-reported physical and mental wellness). Health covariates are divided amongst four components: health behaviors, clinical care, social and economic factors, and the physical environment [14]. CHR&R compiles data from various sources, including the National Center for Health Statistics, the Behavioral Risk Factor Surveillance System, the Centers for Medicare & Medicaid Services, among other data sources to rank U.S counties [17].

Each of the CHR&R covariates are calculated by CHR&R every year with two exceptions. Measures based on vital statistics data and Behavioral Risk Factor Surveillance System survey data are calculated at the National Center for Health Statistics and other units of the Centers for Disease Control and Prevention, and the health care quality measures by the authors of the Dartmouth Atlas of Healthcare [14]. CHR&R covariates are based on different scales (percentages, rates, and averages of survey responses or other metrics). Each factor is standardized individually within each state to the average of each county with a lag of up to 4 years to the publishing of the data. To this end, mortality data associated with COVID-19 pandemic was not available CHR&R at the time of this study and, therefore, not included in the trend analysis. More information on CHR&R data documentation can be found at the CHR&R website [17].

### Outcome variables and covariates

This study mirrored the CHR&R population health approach to study the longitudinal trends for 10 years of data, from 2011 to 2020, of two CHR&R length of life health outcomes in NYS counties: Years of potential life loss before age 75 per 100,000 population (age-adjusted premature death), and premature Age-Adjusted Mortality (number of deaths among residents under age 75 per 100,000 population (age-adjusted) [18].

As per data documentation of CHR&R, Years of Potential Life Lost (YPLL) is a widely used measure of the rate and distribution of premature mortality that focuses on deaths that might have been prevented. YPLL emphasizes deaths of younger persons, whereas statistics that include all mortality are dominated by deaths of the elderly. For example, using YPLL-75, a death at age 55 counts twice as much as a death at age 65, and a death at age 35 counts eight times as much as a death at age 70. Premature Age-Adjusted Mortality was measured as the number of deaths among residents under age 75 per 100,000 population (age-adjusted) [18].

### SDOH covariates

This study preselected covariates to the outcome variable using the following inclusion criteria: variables representative of a SDOH framework with emphasis on behavioral, social economical, clinical, physical environment, race/ethnicity, and rural covariates available for most of the years across a 10 years timespan for all the 62 counties in NYS. As a result, 19 covariates were included in the study and analyzed for the period of 2011–2020.

Table 1 describes 2020 County Health Rankings: measures, data sources, and years of data available.

**Table 1:** County Health Rankings: measures, data sources, and Years of data (2020)

Focus Area	Measure	Description	Source	Year(s)
*Outcome variables*				
**Length of Life**	Years of potential life loss/ Premature death^a^	Years of potential life lost before age 75 per 100,000 population (age-adjusted)	National Center for Health Statistics—Mortality Files	2016–2018
	Premature Age-Adjusted Mortality	Number of deaths among residents under age 75 per 100,000 population (age-adjusted)	National Center for Health Statistics—Mortality Files	2016–2018
*Covariates*				
**Tobacco Use**	Adult smoking	Percentage of adults who are current smokers	Behavioral Risk Factor Surveillance System	2017
**Diet and Exercise**	Adult obesity	Percentage of the adult population (age 20 and older) that reports a body mass index (BMI) greater than or equal to 30 kg/m2	United States Diabetes Surveillance System	2016
	Physical inactivity	Percentage of adults age 20 and over reporting no leisure-time physical activity	United States Diabetes Surveillance System	2016
	Access to exercise opportunities	Percentage of the population with adequate access to locations for physical activity	Business Analyst, Delorme map data, ESRI, & US Census Tigerline Files	2010 & 2019
**Access to Care**	Uninsured	Percentage of the population under age 65 without health insurance	Small Area Health Insurance Estimates	2017
	Primary care physicians	The ratio of population to primary care physicians	Area Health Resource File/American Medical Association	2017
**Education**	High school graduation	Percentage of the ninth-grade cohort that graduates in four years	New York State Education Department	2016–2017
	Some college	Percentage of adults ages 25–44 with some post-secondary education	American Community Survey, 5-year estimates	2014–2018
**Employment**	Unemployment	Percentage of the population ages 16 and older unemployed but seeking work	Bureau of Labor Statistics	2018
**Income**	Median Household Income	The ratio of household income at the 80th percentile to income at the 20th percentile	American Community Survey, 5-year estimates	2014–2018
**Family and Social Support**	Children in poverty^a^	Percentage of people under age 18 in poverty	Small Area Income and Poverty Estimates	2018
	Children in single-parent households	Percentage of children that live in a household headed by a single parent	American Community Survey, 5-year estimates	2014–2018
**Community Safety**	Violent crime	The number of reported violent crime offenses per 100,000 population	Uniform Crime Reporting—FBI	2014&2016
**Air and Water Quality**	Air pollution—particulate matter^+^	The average daily density of fine particulate matter in micrograms per cubic meter (PM2.5)	Environmental Public Health Tracking Network	2014
**Housing and Transit**	Severe housing problems	Percentage of households with at least 1 of 4 housing problems: overcrowding, high housing costs, lack of kitchen facilities, or lack of plumbing facilities	Comprehensive Housing Affordability Strategy (CHAS) data	2012–2016
**Socio-demographic**	Percent Rural	Percentage of the population living in a rural area	Census Population Estimates	2018
	Percent African American	Percent of population	Census Population Estimates	2018
	Percent Asian	Percent of population	Census Population Estimates	2018
	Percent Hispanic	Percent of population	Census Population Estimates	2018

^a^Indicates subgroup data by race and ethnicity is available

### Regression model

A weighted mixed regression model was used to analyze the longitudinal trend in health outcomes as a function of the time-varying covariates listed in Table 2. The intercept was treated as a random effect to account for the within-county correlation (i.e., random intercept model). Standard errors (SE) for the outcomes were estimated from 95% confidence intervals provided in CHR&R data and used to form inverse-variance weights for the regression analysis, giving larger weight to counties with greater precision of estimation. The initial model included terms for year, the 19 covariates, and the two-way interactions between the covariates and year. A preliminary check of the distribution of the outcomes showed that the normal distribution assumption of the mixed model was appropriate.

**Table 2:** Descriptive statistics^a^ of SDOH covariates

	N	Mean	SD	Q1	Median	Q3	Range
Percent Adults with obesity	620	28·13	3·74	26	28	30	15—39
Percent Physically Inactive	558	24·8	3·05	23	25	27	16—33
Percent smokers	605	18·6	4·67	15	18	21	9—33
Percent Adults with Diabetes	620	9·68	1·38	9	10	10	5—16
Percent High School rate	429	82·39	5·91	79	83	86	56—96
Percent with some college	620	60·78	8·24	54·8	60	65·7	41.4—84
Median Household Income	620	54·44	13·51	45·96	50·4	57·1	32·1—115·3
Percent Children in Poverty	620	20·2	5·93	17	20	23	5—44
Percent Single Parent household	620	32·54	7·29	28	33	37	14—64
Percent unemployed	620	6·8	1·83	5·2	6·8	8·2	3·3—12·8
Violent crime rate	614	22·07	14·16	12·9	17·7	25·9	4·1—63·3
Percent Rural	620	44·38	27·29	22·3	50·4	64·2	0—100
Average Daily air pollution	496	10·07	1·64	8·5	10·4	11·3	6·6—13·4
Percent living in severe housing conditions	434	16·97	5·54	14	16	18	9—39
Percent African American	620	6·08	6·44	1·7	4	8·3	0·7—43
Percent Asian	620	2·8	3·99	0·7	1·2	3·4	0·2—27·1
Percent Hispanic	620	7·34	8·88	2·4	3·6	7·8	1—56·4
Percent Uninsured	620	10·3	4·11	7	10	12	4—32
Primary Care Physicians	496	62·17	28·85	42	56	76	7—148

^a^Descriptive statistics calculated across all years and counties (without weighting). SD: standard deviation, Q1: 1^st^ quartile, Q3: 3^rd^ quartile

Median household income: thousands of dollars. Average daily air pollution: average daily amount of fine particulate matter in

micrograms per cubic meter. Violent crime rate: number per 10,000 population. Primary care physician rate: number per 100,000 population

To minimize overfitting, a statistical stepwise backward elimination procedure was performed to obtain an optimal reduced model by minimizing the Bayesian Information Criteria (BIC) [19]. When performing model reduction, the main effect term for the covariate was coupled with the interaction term such that both terms either remained or were eliminated. Consequently, in keeping with the focus on longitudinal effects of the covariates, every covariate in the final, reduced model was accompanied by its interaction term. Inclusion of the interaction terms allows the relationship between a covariate the outcome to vary over time.

To explore implications of the final model, the 62 counties were clustered according to the trend over time in the covariates. For each of the 19 covariates, a linear trend over time was fit separately for each county, resulting in a county-specific intercept/slope pair for the covariate. These county-specific intercepts and slopes were submitted to a k-means clustering algorithm that produced four clusters of counties, where, by design, counties within a cluster were similar with respect to the longitudinal trends in the covariates, and dissimilar to counties in other clusters; the number of clusters was chosen based on ease of interpretation [20]. Intercepts and slopes from the cluster-specific centroids (i.e., average intercept and average slope, calculated, for each risk factor, across members of the cluster) were used to model the linear trend over time for the covariates. These trends were entered into the regression formula from the final model to obtain, for each cluster, the predicted longitudinal trend for the outcome.

All statistical analyses were produced using R version 4.0.4 [21]. The repeated measure analyses were performed with the lme4 package [22].

## Results

Table 2 presents summary statistics for the covariates pooled across all NYS counties for the 10 years studied. Key descriptive findings include: 82% of the population completed high school, 60% have some college, and 62% have access to a primary health physician. Also, 28% of adults are obese, 24% are inactive, and 20% of the children live in poverty.

**Fig. 1:**
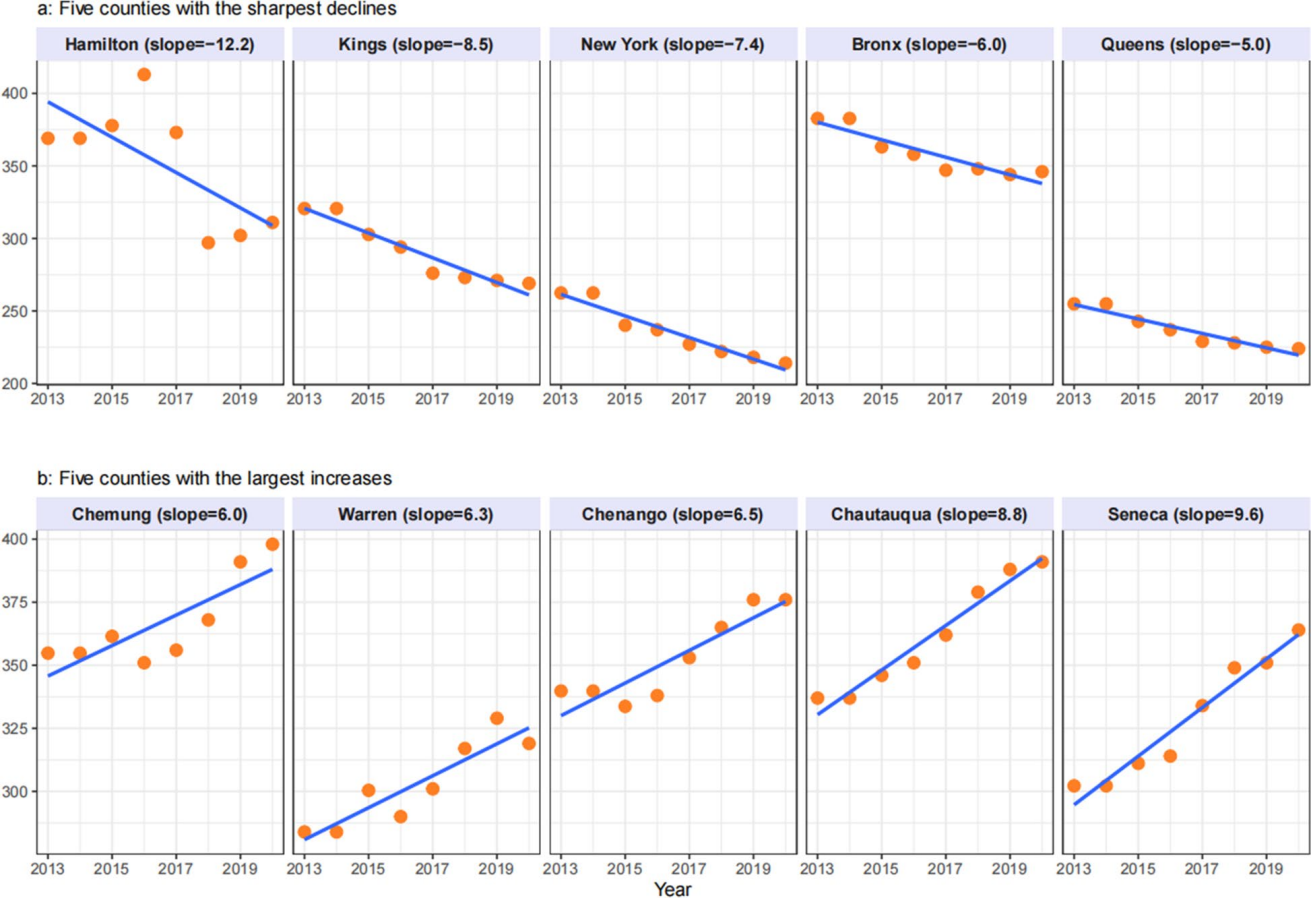
**a**−**b** Linear trend in Premature Mortality over 2011−2020, top increasing and decreasing NYS counties

**Fig. 2:**
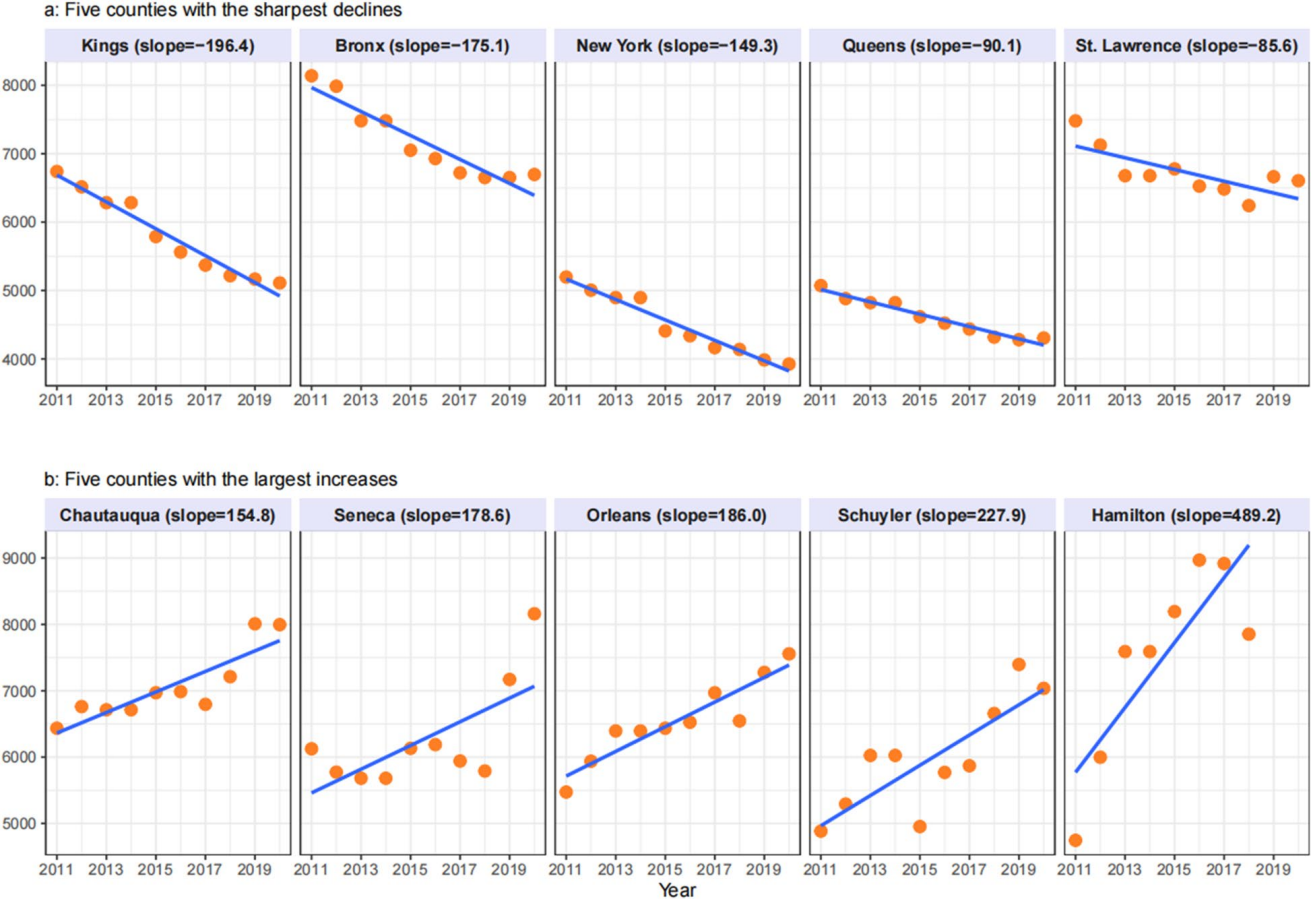
**a**−**b** Linear trend in YPLL over 2011−2020, top increasing and decreasing NYS counties

Figure 1a and b illustrate trends over time for the five counties with the greatest decreases over time (i.e., smallest slopes) and the five counties with the largest increases (i.e., largest slopes) for premature mortality, and in Fig. 2a and b for YPLL.

Findings suggest that Hamilton and four New York City counties (Kings/Brooklyn, New York/Manhattan, Bronx and Queens) have the greatest reductions in premature mortality with slopes ranging from -12·2 to -5·0. Seneca, Chautauqua, Chenango, Warren, Chemung are counties with an increasing trend in premature mortality with slopes from 6·0 to 9·6. Similar to findings in premature mortality, Brooklyn/Kings, Bronx, Manhattan/New York and Queens are counties with downward trends for loss in years of potential life; Hamilton, Schuyler, Orleans, Seneca and Chautauqua are counties with increasing trends in YPLL.

### Multiple regression analysis of the health outcomes

The terms remaining in the model following backward elimination were largely related to the place where people live, which included median household, percent in poverty, percent single parent households, and behavioral covariates, such as high school graduation rate, percent with some college, percent unemployed and percent smokers. Only one factor related to race and ethnicity remained for age-adjusted premature death (percent Asian). All other race and ethnicity covariates (percent Hispanic, African American and Asian) survived elimination to predict YPLL, as well as percent single households, with some college, unemployed, and adults with obesity. For both health outcomes, the health care covariates (primary care physicians’ rate, percent uninsured) were eliminated from the model. The covariates that remained in the models were based on BIC, not on *p*-values. See supplementary table and footnotes for details on the final regression model.

### Predicted trends in the covariates for the County Clusters

The implications of the regression model were explored via predicted longitudinal trends in the outcomes within clusters of counties. The k-means clustering algorithm was applied to the linear trend over time (intercepts & slopes; values not shown) for the 19 covariates. The members of each cluster are displayed in Table 3.

**Table 3:** County Clusters from k-means cluster analysis

Cluster	Cluster Members
1	Allegany, Cattaraugus, Cayuga, Chautauqua, Chenango, Clinton, Columbia, Cortland, Delaware, Essex, Franklin, Fulton, Genesee, Greene, Hamilton, Herkimer, Jefferson, Lewis, Livingston, Madison, Orleans, Oswego, Schoharie, Schuyler, Seneca, St. Lawrence, Steuben, Sullivan, Tioga, Washington, Wayne, Wyoming, Yates
2	Broome, Chemung, Dutchess, Erie, Montgomery, Niagara, Oneida, Onondaga, Ontario, Orange, Otsego, Putnam, Rensselaer, Saratoga, Schenectady, Suffolk, Tompkins, Ulster, Warren
3	Albany, Monroe, Nassau, Manhattan/New York, Staten Island/Richmond, Rockland, Westchester
4	Bronx, Kings/Brooklyn, Queens

**Fig. 3:**
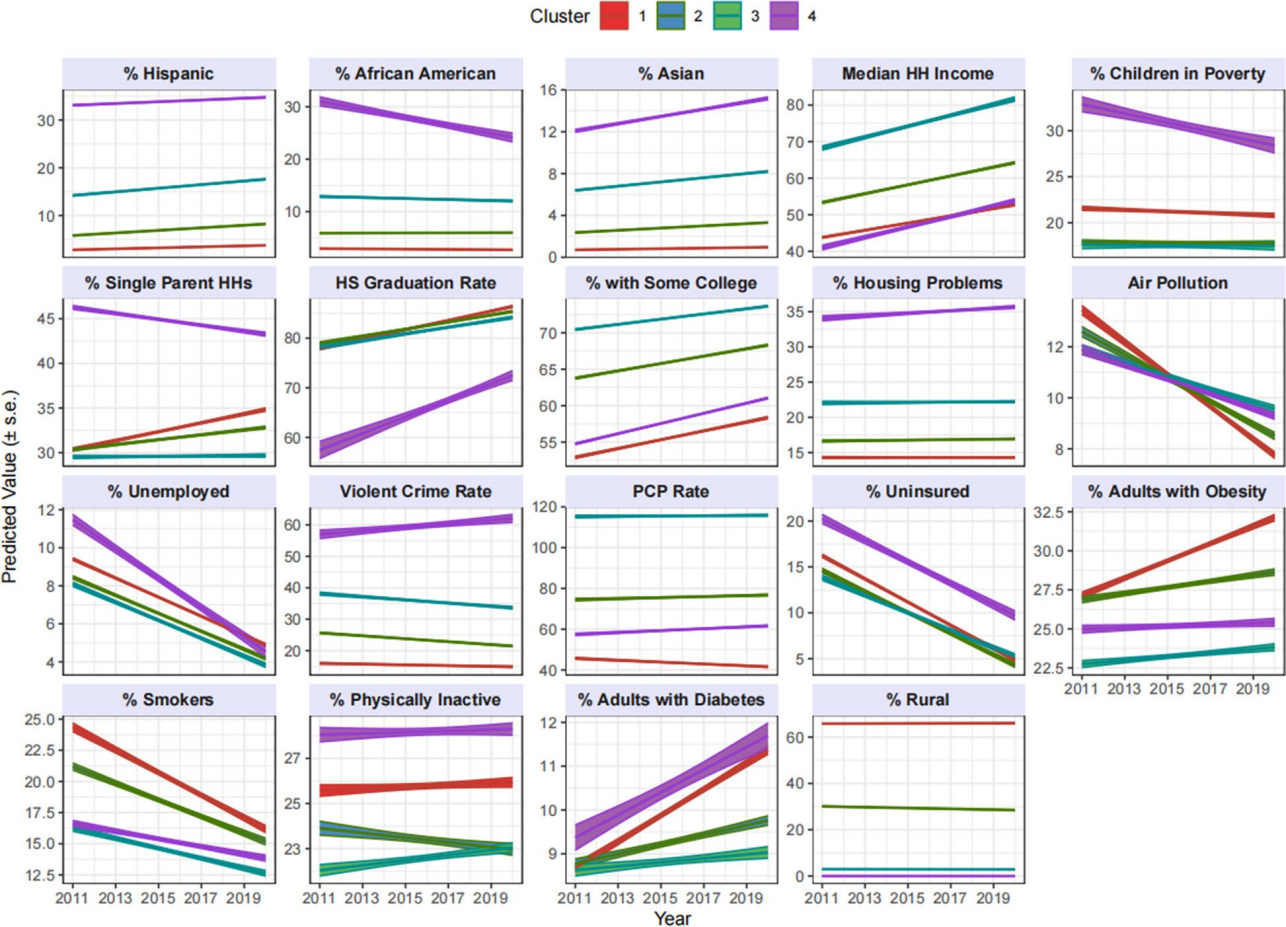
Predicted Trend in Risk Factors over 2011−2020, using Cluster Centroids

Figure 3 displays the predicted linear trend over time (using the centroids), by cluster, for each of the 19 covariates. The interpretation is based on the centroids of each cluster, which indicate the typical linear trend in covariates for counties within the cluster. Cluster 1, which represents 33 of the 62 counties in NYS, contains the most rural counties and the least racially and ethnically diverse counties. Longitudinal trends for this cluster suggest a downward trend in air pollution, unemployment and population uninsured and an upward trend for adults with obesity and diabetes. Clusters 2 and 3 mirror each other in most covariates, with the exception of % rural population. Cluster 4 is comprised of 3 counties (Bronx, Kings/Brooklyn, Queens) represent the most urban and racial and ethnic diverse counties in the state. Counties in this cluster suggest a downward trend in covariates such as percent of: children under poverty; single-headed households; unemployment; population uninsured; and an upward trend in covariates related to educational attainment. Longitudinal trends of concern for cluster 4 include severe housing conditions, violent crime, and percent of population with diabetes.

Noticeable differences across clusters in the trends over time occurred for percent of children in poverty (flat for clusters 1–3, but decreasing for cluster 4), percent of single parent households (flat or increasing for clusters 1–3, but decreasing for cluster 4), percent of adults with obesity (sharpest increase in cluster 1), percent of adults with diabetes (increasing at a faster rate for clusters 1 and 4), and percent of smokers (decreasing at a sharper rate in clusters 1 and 2).

### Predicted trends in health outcomes for County Clusters

**Fig. 4:**
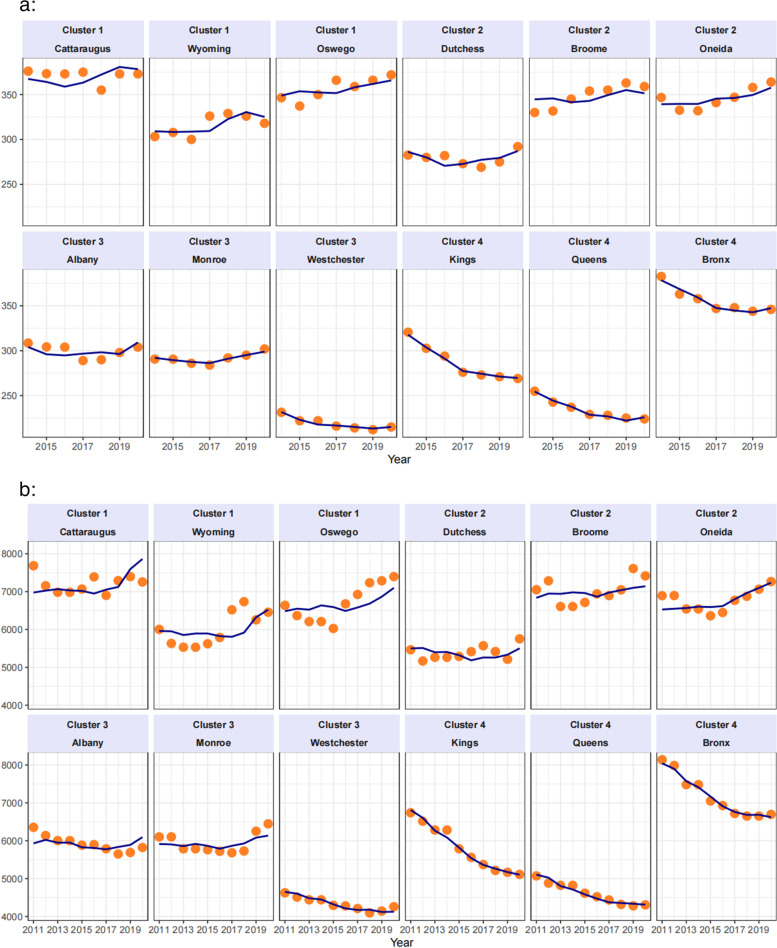
**a** Observed and Predicted Values over 2011−2020 for Premature Mortality for the 3 Counties Closest to Cluster Centroids. **b** Observed and Predicted Values over 2011−2020 for YPLL for the 3 Counties Closest to Cluster Centroids

Predicted values from the final regression model are presented for three counties from each cluster for premature mortality and YPLL in Fig. 4a and b, respectively; the counties were selected as the three counties with covariate trends closest to the cluster centroids.

**Fig. 5:**
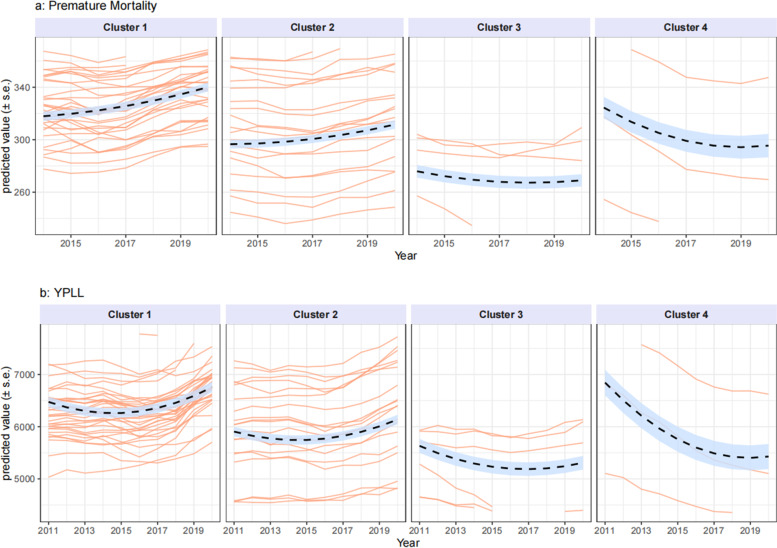
**a**−**b** Predicted Health Outcomes, evaluated at cluster centroids. Orange lines are individual county predictions

Figure 4a indicates that NYC counties Brooklyn/Kings, Queens, and Bronx from cluster 4 have sharply decreasing longitudinal trends in premature mortality controlling for covariates; in contrast, Cattaraugus, Wyoming and Oswego counties from cluster 1 have relatively sharp increasing trends, even after controlling for the effect of the covariates. Similar trends in Fig. 4b are evident for YPLL. Figure 5a-b provides further characterization of the final regression model for premature mortality and YPLL. These figures show the predicted values for each cluster, evaluated using the factor trends in Fig. 4. The typical pattern for counties in cluster 1 is an increasing trend in premature mortality, and those counties in cluster 4 tend to have a downward trend. Clusters 2 and 3 show trends in between those for clusters 1 and 4. These results show how counties can be grouped into clusters on the basis of trends over time in covariates to examine trends in outcomes within each cluster.

## Discussion

This project examined the trend over time, from 2011 to 2020, in health outcomes (age adjusted premature mortality rate and years of potential life loss, YPLL) as a function of trends over time in covariates in New York State (NYS) counties. Differing longitudinal trends in the health outcomes are also evident in the predictions for the four clusters, which were designed to represent the full spectrum of covariates’ trends in NYS.

The CHR&R categorization of counties in 2023 goes from healthiest to least healthy, placing counties in ranking within categories from highest (75–100%), higher (50–75%), lower (25–50%), and lowest (0–25%). The clusters formed in this study from longitudinal trends coincide roughly within these categories, with notable exceptions. For example, counties in cluster 1, such as Allegany, Genesee, Clinton, and Delaware, ranked within the 25–50% category of CHR&R rankings, and this study found they shared similar trends on the health outcomes when studied longitudinally. The New York City (NYC) counties New York/Manhattan (#7), Kings/Brooklyn (#22), and Queens (#12) were ranked among the healthiest counties in New York State (Highest 75%-100%) by the CHR&R 2023 ranking, while the Bronx (#62) ranked as the least healthy county in NYS [15]. Conversely, this study grouped the Bronx with Kings/Brooklyn and Queens into cluster 4 and New York/Manhattan and Staten Island/Richmond into cluster 3 due to similar trends over time in the covariates.

CHR&R’s goal is to highlight the importance of geographical location and health outcomes and identify where disparities exist within every state and county [18]. CHR&R consistently ranked the Bronx from 2010 to 2023 among the least healthy counties in NYS, whereas this study suggests the Bronx is altering the historical trend compared with other counties in the state by having sharply decreasing longitudinal trends in premature mortality controlling for covariates. The difference may be explained by the focus in this study on modelling longitudinal trends in health outcomes (from 2011 to 2020) based on longitudinal trends in the covariates, while CHR&R ranks counties uses cross sectional estimates for each year. CHR&R for the year 2020 did not include COVID-19 related mortality and therefore, the decreasing trend in premature mortality was not included. According to CHR&R documentation, COVID-19 related mortality was included in CHR&R 2023 rankings [18]. More research is needed to explore the impact of COVID-19 on the covariates and if these would alter the trends on the health outcomes on length of life.

Key implications of the model presented in this research are in the prediction of the longitudinal trends in the outcomes. The modeling of interactions allowed the relationship between the covariates versus health outcomes to vary over time, as opposed to assuming a static relationship over time. Findings from this study present an opportunity to have a baseline of where the county has been and where it can go in each of the covariates modelled and in relation to the health outcomes.

The findings from the cluster analyses suggests that the counties in cluster 4, which contains three of the five NYC counties, have a positive outlook. For example, percent of children in poverty, single-family households, and percent of unemployed are declining while at the same time, high school graduation trends, percent with some college, and median household income have an increasing trend. The covariates that remain a concern with an increasing trend longitudinally are violent crime rates and percent of adults with diabetes. According to data from the New York Police Department (NYPD), homicides and firearm injuries have increased from 2019 to 2020 in NYC and 26% of the homicides, 23% of the rapes, 26% of robberies, and 28% of the felonious assaults occur in the Bronx [23]. The vast majority of fatal and non-fatal injuries were males from an ethnic and racial minority [23]. When it comes to adults with diabetes, research suggest that in NYC more than a quarter of those diagnosed are Hispanic and represent the fourth cause of death in all NYC counties [24]. The current analysis suggests that the percent of Hispanics and the percent of Asian are increasing over time, while the percent of African American/Black is decreasing in NYC. Policy-makers need to account for demographic and factor trends influencing health outcomes.

On the other hand, the cluster analysis suggests a complicated trend for counties in Clusters 1 and 2. The counties in these clusters are those with the lowest percentages of racial and ethnic minorities and increasing trends of obesity, diabetes, and single-headed household, while showing the steepest upward trend in premature mortality. These findings are aligned with health disparities research in rural areas. Mortality rates in rural areas have improved at a slower pace compared to improvements in urban areas of the United States [25]. Furthermore, the top five illnesses of age-adjusted premature morality are heart disease, cancer, unintentional injury, chronic lower respiratory disease, and stroke [26]. The causes of this excess mortality is not straightforward. Research suggests differences in access to health care services, a relation between unemployment rates and high school graduation rates and chronic diseases, and socioeconomic covariates can only explain partially variations in age-adjusted premature mortality rates across rural counties [27–29]. Findings from this study cannot make generalizable statements of the specific causes of rural health disparities in NYS, and therefore, more research is needed in this area.

## Limitations

Limitations to this study are categorized into issues related to the data, and limitations in the interpretation of results. The CHR&R project compiles health outcome and associated covariates from a variety of national sources with minimal missing data. However, the changing definition over time for some of the covariates (e.g., high school graduation rate) meant that some years were excluded from the analysis for a small number of covariates. In addition, the analysis did not separate out minorities (e.g. American Indian and Alaska Natives) for which the data were too limited to make predictions.

This study analyzed CHR&R available data from the 2011–2020 rankings. Each variable is a compilation of different estimates (e.g. percentages, rates, averages) and then individualized standardized within the state to the average of each county with a lag of up to 4 years to the publishing of the data. This meant that each health outcome and covariate was lagged 2–4 year of past data. Therefore, the interpretation of results does not include COVID-related mortality as that data were not available at the time of this study.

## Conclusions

This study analyzed the longitudinal trends in NYS counties of age-adjusted premature mortality rate and years of potential life loss rate (YPLL) from 2011–2020 using County Health Ranking and Roadmaps (CHR&R) data to identify similarities and trends among the counties in the state. The analysis clustered counties according to the longitudinal trends of the covariates, and by doing so identified clusters of counties that shared similar trends among the covariates, to later examine trends in the health outcomes through a regression model. The strength of this approach lies in the predictive feature of what is to come for the counties by understanding the covariates and set prevention goals. Therefore, the results from this study are a starting point to further investigate the specifics within these counties and call for dialogue and coordinated efforts to improve this population’s health outcomes.

## Supplementary Information

**Additional file 1: Supplementary Table 1.** Regression coefficients from final regression model for age-adjusted premature mortality rate and YPLL rate in NYS counties (2011-2020).

## Data Availability

This is a secondary data analyses project using data from the County Health Rankings and Roadmaps CHR&R data set. The analyses generated are included in this published article and its supplementary information files.
