# Supplementary material for: Longitudinal trends (2011–2020) of premature mortality and years of potential life loss (YPLL) and associated covariates of the 62 New York State counties
Source: Int J Equity Health. 2023 May 16;22:89. doi: 10.1186/s12939-023-01902-w (PMC10188228; doi:10.1186/s12939-023-01902-w)
Supplement: Supplementary file 1 — Additional file 1: Supplementary Table 1. Regression coefficients from final regression model for age-adjusted premature mortality rate and YPLL rate in NYS counties (2011-2020). [file 12939_2023_1902_MOESM1_ESM.docx]

| **Regression coefficients from final regression model for age-adjusted premature mortality rate and YPLL rate in NYS counties (2011-2020)** | | | | | | |
| --- | --- | --- | --- | --- | --- | --- |
|  |  |  |  |  |  |  |
|  | **Premature Mortality** | | | **YPLL** | | |
| **Model Term** | **Estimate ~ ^** | **SE** | **p-value** | **Estimate ~ *** | **SE** | **p-value** |
| Intercept | 300·24 | 3·00 | <0·001 | 5948·77 | 71·82 | <0·001 |
| Year | 5·06 | 0·69 | <0·001 | 30·97 | 10·35 | 0·003 |
| Percent Hispanic | - | - | - | 6·41 | 9·74 | 0·512 |
| Percent African American | - | - | - | -11·30 | 7·29 | 0·122 |
| Percent Asian | -3·71 | 0·69 | <0·001 | -30·95 | 17·20 | 0·074 |
| Median Household Income | -0·31 | 0·20 | 0·121 | - | - | - |
| Percent Children in Poverty | 0·81 | 0·27 | 0·003 | - | - | - |
| Percent Single Parent Households | 0·54 | 0·29 | 0·069 | 26·64 | 6·21 | <0·001 |
| High School Graduation Rate | -1·16 | 0·23 | <0·001 | - | - | - |
| Percent with Some College | -0·56 | 0·32 | 0·079 | -15·78 | 7·37 | 0·033 |
| Percent Unemployed | 4·54 | 0·80 | <0·001 | 59·76 | 12·84 | <0·001 |
| Percent Adults with Obesity | - | - | - | 13·63 | 5·86 | 0·020 |
| Percent Smokers | -0·43 | 0·20 | 0·029 | - | - | - |
| Percent Rural | - | - | - | 4·94 | 3·79 | 0·196 |
| Year x Percent Hispanic | - | - | - | -1·05 | 0·50 | 0·035 |
| Year x Percent African American | - | - | - | -5·51 | 0·62 | <0·001 |
| Year x Percent Asian | 0·01 | 0·04 | 0·796 | -0·26 | 0·65 | 0·693 |
| Year x Median Household Income | -0·06 | 0·02 | 0·001 | - | - | - |
| Year x Percent Children in Poverty | -0·18 | 0·05 | <0·001 | - | - | - |
| Year x Percent Single Parent Households | 0·12 | 0·03 | <0·001 | 1·11 | 0·46 | 0·016 |
| Year x High School Graduation Rate | 0·13 | 0·06 | 0·034 | - | - | - |
| Year x Percent with Some College | -0·11 | 0·02 | <0·001 | -1·95 | 0·77 | 0·012 |
| Year x Percent Unemployed | -1·65 | 0·19 | <0·001 | -26·23 | 2·92 | <0·001 |
| Year x Percent Adults with Obesity | - | - | - | 7·89 | 1·19 | <0·001 |
| Year x Percent Smokers | 0·58 | 0·09 | <0·001 | - | - | - |
| Year x Percent Rural | - | - | - | -1·16 | 0·29 | <0·001 |
| ~Random intercept model. Year was centered at 2015 and the risk factors were mean-centered. SE: standard Error. | | | | | |  |
| ^ BIC=3515·4 for the full model; BIC=3452·1 for the reduced model· | | | | | |  |
| * BIC=6370·7 for the full model; BIC=6281·5 for the reduced model. | | |  |  |  |  |

*Note*: Although p-values are listed above for the final model, model reduction was performed according to BIC criteria, without any reference to p-values.
